# Reconstruction of Dispersal Patterns of Hypervirulent Meningococcal Strains of Serogroup C:cc11 by Phylogenomic Time Trees

**DOI:** 10.1128/JCM.01351-19

**Published:** 2019-12-23

**Authors:** Alessandra Lo Presti, Arianna Neri, Cecilia Fazio, Paola Vacca, Luigina Ambrosio, Clara Grazian, Brunero Liseo, Giovanni Rezza, Martin Christopher James Maiden, Paola Stefanelli

**Affiliations:** a Department of Infectious Diseases, Istituto Superiore di Sanità, Rome, Italy; b Department of Economics, Università degli Studi “G. d’Annunzio,” Chieti, Italy; c School of Mathematics and Statistics, University of New South Wales, Sydney, Australia; d “Sapienza Università di Roma, MEMOTEF,” Rome, Italy; e Department of Zoology, University of Oxford, Oxford, United Kingdom; Brigham and Women’s Hospital

**Keywords:** *Neisseria meningitidis*, public health surveillance, invasive meningococcal disease, phylogeography

## Abstract

Neisseria meningitidis is one of the few commensal bacteria that can even cause large epidemics of invasive meningococcal disease (IMD). *N. meningitis* serogroup C belonging to the hypervirulent clonal complex 11 (cc11) represents an important public health threat worldwide. We reconstructed the dispersal patterns of hypervirulent meningococcal strains of serogroup C:cc11 by phylogenomic time trees. In particular, we focused the attention on the epidemic dynamics of C:P1.5.1,10-8:F3-6;ST-11(cc11) meningococci causing outbreaks, as occurred in the Tuscany region, Italy, in 2015 to 2016. A phylogeographic analysis was performed through a Bayesian method on 103 Italian and 208 foreign meningococcal genomes. The C:P1.5.1,10-8:F3-6;ST-11(cc11) genotype dated back to 1995 (1992 to 1998) in the United Kingdom. Two main clades of the hypervirulent genotype were identified in Italy. The Tuscany outbreak isolates were included in different clusters in a specific subclade which originated in the United Kingdom around 2011 and was introduced in Tuscany in 2013 to 2014. In this work, phylogeographic analysis allowed the identification of multiple introductions of these strains in several European countries and connections with extra-European areas. Whole-genome sequencing (WGS) combined with phylogeography enables us to track the dissemination of meningococci and their transmission. The C:P1.5.1,10-8:F3-6;ST-11(cc11) genotype analysis revealed how a hypervirulent strain may be introduced in previously naïve areas, causing a large and long-lasting outbreak.

## Abstract (precis)

Neisseria meningitidis is one of the few commensal bacteria that can even cause large epidemics of invasive meningococcal disease (IMD). *N. meningitis* serogroup C belonging to the hypervirulent clonal complex 11 (cc11) represents an important public health threat worldwide. We reconstructed the dispersal patterns of hypervirulent meningococcal strains of serogroup C:cc11 by phylogenomic time trees.

## INTRODUCTION

Invasive meningococcal disease (IMD) is a severe, life-threatening condition caused by Neisseria meningitidis that leads to more death and disability among infants and adolescents than any other microbial infection (1). The pathogen is classified into 12 capsular serogroups; in Europe, four of these groups (serogroups B, C, W, and Y) are most commonly associated with IMD, which can take the form of septicemia, meningitis, or both (2). Meningococcus is a genetically highly diverse bacterium which is mostly carried asymptomatically in the oropharynx of healthy individuals and transmitted from person to person via the aerosol route. While invasion is a rare event compared to carriage (3), some genotypes of the meningococcus, recognized as clonal complexes (ccs) by multilocus sequence typing (MLST) (4), are much more likely to cause IMD than others (5). These are referred to as the “hypervirulent lineages” (6).

In Italy, the incidence of IMD is among the lowest in Europe, with a rate of 0.33 per 100,000 in 2017 (7). Vaccination against N. meningitidis serogroup C (MenC), which was the most commonly reported cause of IMD in the country at the time, was introduced in several regions in 2005 to 2006, as recommended immunization of children at 13 months of age, but vaccination was introduced at the national level only in 2012 in accordance with the national immunization plan (8). After the introduction of vaccination on the national level, the proportion of MenC IMD cases in Italy declined (7). During the period from 2010 to 2015, all the Italian regions showed stable low incidence of IMD, with the exception of the Tuscany region, where an increase of cases due to MenC was reported starting in the year 2015 (9). The outbreak lasted for 2 years, in 2015 and 2016, causing a total of 62 cases of IMD, and was attributable to MenC strains belonging to the hypervirulent cc11 complex, with the finetype C:P1.5-1,10-8:F3-6:ST-11(cc11), which underwent a clonal expansion in the area (10). In Italy, clusters due to this MenC strain had previously been described in 2008 and in 2012 (11, 12); however, none of these caused a large epidemic event with sustained transmission, such as the outbreak that occurred in Tuscany in 2015 to 2016. Since the epidemic dynamics of MenC hypervirulent strains, and in particular those of C:P1.5-1,10-8:F3-6:ST-11(cc11) meningococci, remain poorly defined, hereby, we reconstructed the dispersal patterns of hypervirulent meningococcal strains of C:P1.5-1,10-8:F3-6:ST-11(cc11) by a phylodynamic approach, modeling genetic sequence data from the outbreak isolates and those available in the http://pubmlst.org/neisseria database (accessed June 2017), to understand how the strain may be introduced and spread across countries.

## MATERIALS AND METHODS

### Invasive meningococcal disease surveillance.

In Italy, all IMD cases are reported to the Ministry of Health and the Italian Institute of Public Health (Istituto Superiore di Sanità [ISS], http://old.iss.it/mabi/, in the context of the National Surveillance System, which is coordinated by the ISS, which acts as the National Reference Laboratory (NRL) for IMD. The Italian case deﬁnition of IMD is based on the EU Commission Implementing Decision 2012/506/EU of 8 August 2012. For each IMD case, epidemiological information is routinely collected by the NRL and managed using a dedicated database. All available samples (cultivated isolates, blood, or cerebrospinal ﬂuid) are sent to the NRL for laboratory conﬁrmation and further microbiological characterization.

### Microbiological and molecular analyses.

Chromosomal DNA samples from all the MenC collected by the NRL from 1 January 2012 to 31 December 2017 were extracted using the QiAmp DNA minikit (Qiagen, Hilden, Germany), according to the manufacturer’s protocol. Multilocus sequence typing (MLST) and PorA and FetA typing were performed as described at https://pubmlst.org/neisseria. The meningococcal fine type was defined as follows: capsular group: PorA (P1) variable region 1 (VR1), VR2: FetA VR: sequence type (ST) (clonal complex [cc]) (13). Whole-genome sequencing (WGS) was performed using Illumina MiSeq platform (kit v3, 600 cycles), as described previously (9), on 101 viable meningococci with the C:P1.5-1,10-8:F3-6:ST-11(cc11) genotype. In addition, two isolates with genotype C:P1.5-1,10-8:F3-6:ST-2780(cc11) were included in the analysis.

### Isolate sets used for analysis.

The whole data set comprised the genome sequences from 311 N. meningitidis isolates obtained between 1998 and 2017 collected in the PubMLST *Neisseria* database (https://pubmlst.org/neisseria). It included the 103 genomes obtained from N. meningitidis Italian isolates, collected from 2012 to 2017, together with 208 C:P1.5-1,10-8:F3-6:ST-11(cc11) genomes obtained from meningococci isolated from IMD cases in the following countries: United Kingdom (*n* = 128), France (*n* = 30), South Africa (*n* = 20), Spain (*n* = 8), Ireland (*n* = 5), Slovenia (*n* = 5), Sweden (*n* = 5), Iceland (*n* = 4), Malta (*n* = 2), and Canada (*n* = 1) (www.neisseria.org; last accessed 16 June 2017). All 311 genomes were compared gene by gene using the BIGSdb Genome Comparator tool (14). The genomes were analyzed using the core genome MLST (cgMLST) scheme, in the PubMLST *Neisseria* database (https://pubmlst.org/neisseria), and the core genome alignment of the 311 genomes was generated. Two subsets of the whole data set were made. The first subset included only Italian genomes (*n* = 103) and was used to investigate the phylogenetic and epidemiological relationships within Italy. The second subset included 133 N. meningitidis isolates obtained in the period from 2007 to 2017 from Italy (*n* = 63), United Kingdom (*n* = 47), France (*n* = 9), Slovenia (*n* = 5), Sweden (*n* = 4), Ireland (*n* = 3), Malta (*n* = 1), and South Africa (*n* = 1). This subset was used to place the Tuscany outbreak strain (12) into an international context.

### Phylogenetic analysis.

Core genome recombination analysis was performed with BratNextGen (BNG) (15) on the whole data set with the aligned core meningococcal genome to obtain recombination-free input sequences for further phylogenetic analysis as described previously (16–18). The proportion of shared ancestry (PSA) tree cutoffs equal to 0.25 was used. The analysis was conducted with a total of 20 iterations of hidden Markov model (HMM) parameter estimation. Statistically significant (*P* value not exceeding 5%) recombination in the core genome was determined with 100 parallel permutation runs.

Single-nucleotide polymorphisms (SNPs) were derived from the core genome shared by all the isolates. SNPs were exported as variable sites using MEGA removing SNP sites with ambiguities, missing data, and gaps (19). The SNPs associated with recombinogenic regions were removed. Phylogenetic analysis was conducted to reconstruct the relationships among the isolates. Evaluation of the best-fitting model of nucleotide substitution was performed with the JModeltest (20, 21). The phylogenetic signal was investigated by using Treepuzzle (22), with the likelihood mapping analysis, using 10,000 random quartets to obtain a comprehensive picture of the phylogenetic quality and to estimate the amount of phylogenetic information. This analysis indicated <30% of quartets in the star-like region, meaning that sequence evolution conformed to a resolved tree. The Xia’s test of substitution saturation, implemented in DAMBE (23), indicated that the data set was suitable for further phylogenetic analysis, as the nucleotide substitutions were not saturated (see Table S3 in the supplemental material).

To determine the global context of the N. meningitidis isolates from Italy with respect to foreign isolates, a maximum likelihood (ML) tree on the core genome SNP alignment (whole data set) was performed with the IQ-TREE (24), applying the general tree reversible (GTR) substitution model (previously estimated) with ascertainment bias correction (ASC). The statistical support for internal branches of the ML tree was evaluated by bootstrapping (1,000 replicates) and fast likelihood-based sh-like probability (SH-aLRT). The N. meningitidis core genome SNP alignment of the first subset of isolates was analyzed by using Mr Bayes (25). A Markov chain Monte Carlo search was done for 10 × 10^6^ generations using tree sampling every 100th generation, with the GTR substitution model with ASC and a burn-in fraction of 25%. Statistical support for specific clades, subclades, and clusters was obtained by calculating the posterior probability of each monophyletic clade (posterior probability > 0.90), and a posterior consensus tree was generated after a 25% burn-in. In the evolutionary context, the clustering topology was described by specifying clade, subclade, and cluster. A clade was defined as a monophyletic group, meaning a group of taxa sharing a common ancestor. Each clade, which may contain many isolates, can be further subdivided into subclades, according to the tree topology, phylogenetic relationships, and evolutionary bifurcation. Finally, each edge in the tree defines a unique cluster, and sequences that are sufficiently more related to one another than to the rest of the data set can be designated a distinct cluster.

### Time-scaled and phylogeographic analysis.

Linear regression of root-to-tip distances against date of isolation was performed using TempEst (26) to investigate the temporal structure and the correlation between genetic distance against sampling time. The demographic history and phylogeography of the whole data set and the second subset of isolates, including evolutionary rates and date estimates, were estimated with the Bayesian Markov chain Monte Carlo (MCMC) method implemented in BEAST (27), using the GTR substitution model. Alternative clock models, strict and relaxed with an uncorrelated log normal rate distribution, and demographic models, including constant population size, exponential growth, nonparametric smooth skyride plot Gaussian Markov random field (GMRF), and nonparametric Bayesian skyline plot (BSP) were tested on the filtered core genome SNP alignment, accounting for invariant sites. The Bayes factor (BF) (using marginal likelihoods) tests were used to compare the alternative models and to select the best fitting as previously described (28); only values of 2 ln BF of >6 were considered significant. Chains were conducted until convergence was reached and assessed by calculating the effective sampling size (ESS) for each parameter. Only parameter estimates with ESSs of >200 were accepted. Uncertainty in the estimates was indicated by 95% highest posterior density (HPD) intervals. Maximum clade credibility trees (MCCT) were summarized with Tree-Annotator, after a 10% burn-in. Statistical support for specific clade was assessed by posterior probability (pp of >0.90). The continuous-time Markov chain (CTMC) process over discrete sampling locations (BEAST) (27) with Bayesian Stochastic Search Variable Selection (BSSVS) model was used for the phylogeography inferences. Analysis and visualization of the different aspects of the phylogeographic diffusion were performed by using SpreaD (29). The temporal dynamics of the spatial N. meningitidis diffusion were provided by snapshot maps of the dispersal pattern through Google Earth (Google Earth Pro V.7.3.2.5491) (accessed April 2018).

## RESULTS

### Global phylogenetic analysis.

**FIG 1:**
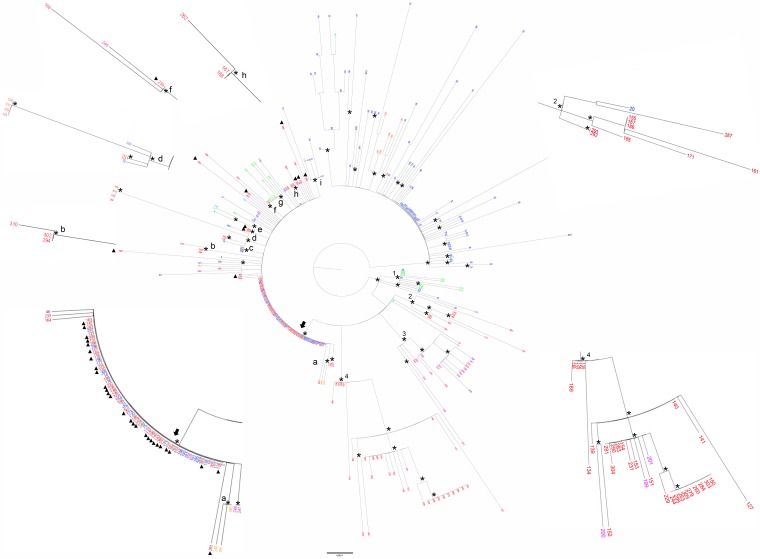
Maximum likelihood phylogenetic tree of N. meningitidis core genome SNP alignment of the whole data set. The tree was midpoint rooted. Branch lengths were estimated with the best-fitting nucleotide substitution model according to a hierarchical likelihood ratio test. The scale bar represents genetic distances based on nucleotide substitutions per SNP site. An asterisk along a branch represents significant statistical support for the clusters subtending that branch (bootstrap support > 90%). The main clades and clusters are highlighted. The colors of the tips represent strains from different locations (United Kingdom, blue; Malta, green; France, fuchsia; Ireland, light blue; Spain, orange; Canada, violet; Italy, red; Slovenia, ochre yellow; South Africa, light green). The subclade of the larger clade is highlighted by an arrow. The small letters indicate the supported clusters located inside this subclade. A full triangle symbol next to the tips indicates “Tuscany-outbreak-strains.” The values around the tree (supported zoomed clusters and area of the subclade) highlight the phylogenetic relationships of the Italian genomes between them and respect to foreign isolates.

ML analysis of Italian isolates versus other isolates (whole data set) showed two main supported clades (Fig. 1). The smaller clade included genomes from the United Kingdom, Ireland, Iceland, Spain, and Malta. The larger clade consisted of four statistically supported clusters (clusters 1 to 4) and a subclade, which included isolates originating in Italy (Fig. 1). Cluster 1 included genomes from South Africa and the United Kingdom, whereas cluster 3 included isolates from France, United Kingdom, Spain, and Sweden. In cluster 2, nine genomes from Italy appeared to be related to an isolate from the United Kingdom, while cluster 4 included three French isolates clustering with 29 isolates from several Italian regions (Fig. 1; see also Table S1 in the supplemental material). Finally, Italian isolates clustered in a subclade with isolates from other countries, including the United Kingdom, France, Sweden, Slovenia, Malta, Ireland, and South Africa (Fig. 1). All genomes belonging to the “Tuscany-outbreak strain” were assigned to this subclade (Fig. 1). Among the statistically supported clusters in this subclade, two of these clusters included only Italian isolates and other two included both Italian isolates and other isolates (Fig. 1, clusters b, d, f, and h). The remaining clusters (Fig. 1, clusters a, c, e, g, and i) comprised only non-Italian isolates.

### Bayesian analysis of N. meningitidis Italian isolates (first subset).

**FIG 2:**
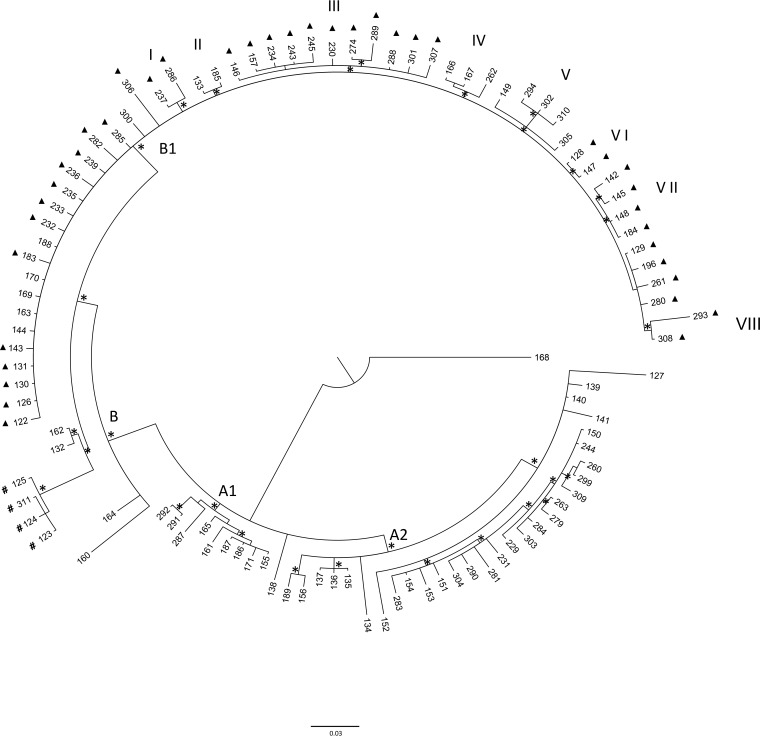
Bayesian phylogenetic tree of the first subset of N. meningitidis core genome SNP alignment. The tree was rooted using the midpoint rooting method. Branch lengths were estimated with the best-fitting nucleotide substitution model according to a hierarchical likelihood ratio test. The scale bar represents genetic distances based on nucleotide substitutions per SNP site. An asterisk along a branch represents significant statistical support for the clade subtending that branch (posterior probability > 90%). A full triangle symbol next to the tips indicates “Tuscany-outbreak-strains.” The four isolates from cases occurred on a cruise ship docked in the port of Livorno in Italy are indicated by a *#* symbol next to the tips. The main clades and clusters are highlighted.

Bayesian analysis of the first subset of isolates (Fig. 2) identified a supported clade, clade A1, which included isolates from various Italian regions that were closely related to each other. Clade A2 included six isolates and a subclade comprising 23 isolates, all originating in different Italian regions (Fig. 2; Table S1). Eight Italian isolates appeared externally located to subclade B1. Among these isolates was a well-supported cluster, which included four isolates from cases that occurred on a cruise ship docked in the port of Livorno, as described previously (30), and two other Italian isolates (2012 to 2013). All the “Tuscany-outbreak strain” isolates were located in the B1 subclade (Fig. 2), most of them in different clusters. Two isolates belonged to cluster I, 11 isolates belonged to cluster III, 2 isolates to cluster VI, 4 isolates to cluster VII, and 2 isolates to cluster VIII. Twenty isolates, 14 of which belonged to the “Tuscany-outbreak strain,*”* were located in subclade B1, but not aggregated in a specific internal cluster. Clusters II, IV, and V comprised other Italian genomes, except those of the “Tuscany-outbreak strain.”

### Bayesian reconstruction of the time-scaled phylogeny and phylogeographic analysis.

The root-to-tip regression analysis of the genetic distances against sampling time produced a correlation coefficient of 0.70, and a coefficient of determination (*R*^2^) of 0.58, indicating a positive correlation, and supporting the suitability of these data for a molecular clock analysis. Formal model selection (BF tests) indicated the relaxed molecular clock and BSP as the most appropriate model for our data (Table S2). The mean evolutionary rates estimated were 3.7 × 10^−6^ substitutions per site per year (95% highest posterior density [HPD] intervals 3.1 × 10^−6^ to 4.4 × 10^−6^) for the whole data set and 3.1 × 10^−6^ (2.4 × 10^−6^ to 3.8 × 10^−6^) for the second subset of isolates.

**FIG 3:**
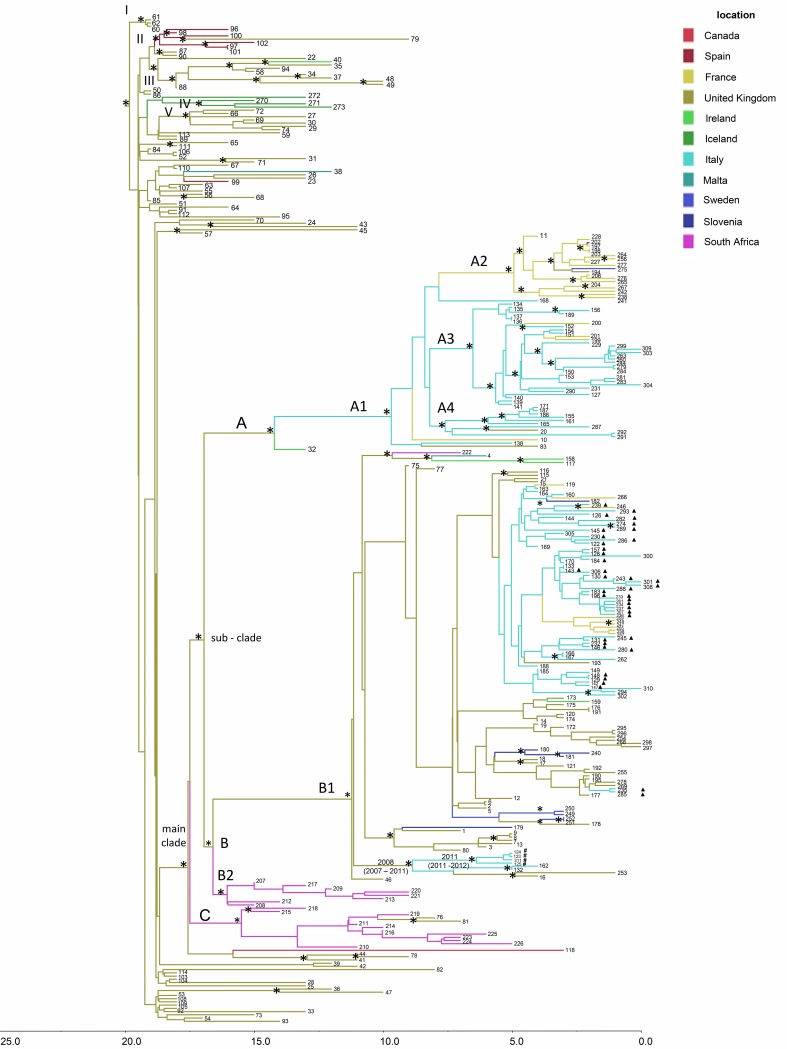
Bayesian phylogeographic tree of N. meningitidis strains of the whole data set. The axis below the tree shows the time (in years) before the present. The main statistically supported clades and clusters are indicated. An asterisk along a branch represents significant statistical support for the clade subtending that branch (posterior probability > 90%). A full triangle symbol next to the tips indicates “Tuscany-outbreak-strains.” The four isolates from cases that occurred on a cruise ship docked in the port of Livorno in Italy are indicated by a *#* symbol next to the tips. Geographic locations are indicated by different colors in the tree and reported in the location key.

The phylogeographic analysis of N. meningitidis strains of the whole data set (Fig. 3) indicated that the root of the tree had a time to the most recent common ancestor (tMRCA) of 22 years, corresponding to the year 1995 (95% HPD, 1992 to 1998) with the most likely location in the United Kingdom (state probability [sp] = 0.97). From this ancestor, five supported clusters (clusters I to V), including only European genomes and a main clade, were identified (Table 1). Inside the main clade, clade A and subclade B1 were defined. Clade A originated in 2002 (95% HPD, 2000 to 2004) in the United Kingdom (sp = 0.93) comprising the subclade A1 that originated in 2007 (95% HPD, 2002 to 2009) in Italy (sp = 0.82). Within subclade A1, some genomes from meningococci in France were closely related to those from Spain, Sweden, and the United Kingdom, forming the supported subcluster A2 (Table 1). The significant subcluster A3, with 3 genomes from France, was related to 29 genomes from Italy. Subcluster A4 included nine Italian genomes related to 1 from the United Kingdom (Fig. 3 and Table 1). The B1 subclade dated back to 2005 (95% HPD, 2002 to 2007), with the most probable origin in the United Kingdom (sp = 0.99). This subclade included all the “Tuscany-outbreak strain” isolates (Fig. 3), together with other genomes from Italy, the United Kingdom, Ireland, France, Slovenia, Sweden, and Malta and one from South Africa. The four isolates collected during the outbreak on the cruise ship clustered in subclade B1, forming a highly statistically supported, distinct cluster dated to 2008 in the United Kingdom (95% HPD, 2007 to 2011). This cluster also included two other genomes from Italy and two from the United Kingdom. Phylogeographic analysis showed the United Kingdom as the most probable origin (sp = 0.6) for this cluster. The four isolates collected during the outbreak on the cruise ship dated back to 2011 in Italy (96% HPD, 2011 to 2012).

**TABLE 1:** Time to the most recent common ancestor for the internal nodes, 95% highest posterior density estimates, the most probable location, and the state probability of the most important clades and clusters (whole data set)

Name^*a*^	tMRCA^*b*^ (yr)	95% HPD^*c*^	Most probable location	State probability
I	1997	1997−1998	United Kingdom	1
II	1998	1997−1999	Spain	0.98
III	1998	1997−1999	United Kingdom	1
IV	2000	1999−2002	Iceland	0.99
V	2000	1999−2001	United Kingdom	1
Main clade	1999	1998−2000	United Kingdom	0.98
Subclade	2000	1998−2001	United Kingdom	0.97
A	2002	2000−2004	United Kingdom	0.93
A1	2007	2002−2009	Italy	0.82
A2	2012	2011−2013	France	0.99
A3	2010	2008−2011	Italy	0.99
A4	2009	2009−2012	Italy	0.99
B	2000	1999−2002	United Kingdom	0.92
B1	2005	2002−2007	United Kingdom	0.99
B2	2001	2000−2002	South Africa	0.99
C	2001	2000−2003	South Africa	0.97

a Clades, subclades, or clusters, as reported in Fig. 3.

b tMRCA, time to the most recent common ancestor.

c HPD, highest posterior density.

### Temporal dynamics of spatial diffusion.

**FIG 4:**
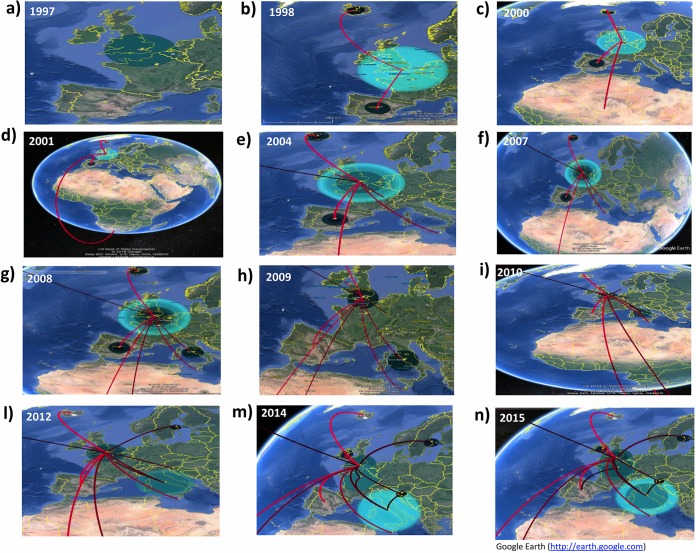
Temporal dynamics of spatial diffusion of N. meningitidis strains (whole data set), obtained from the location annotated MCCT. The snapshots of the main dispersal pattern were reported. The lines connecting different locations represent branches in the MCCT on which state exchanges occur and circle areas reflect the number of branches maintaining a particular state at that time point. The maps were based on satellite pictures made available in Google Earth (Google Earth Pro V.7.3.2.5491).

The temporal dynamics of spatial diffusion suggested that C:P1.5-1,10-8:F3-6:ST-11(cc11) N. meningitidis strains, after accumulating in the United Kingdom (Fig. 4a), by 1998, spread first to Spain and subsequently to Iceland (Fig. 4b). By 2000, these strains reached a more distant location, South Africa, in 2001 (Fig. 4c and d). By 2004, the C:P1.5-1,10-8:F3-6:ST-11(cc11) N. meningitidis appeared to have spread from the United Kingdom to Ireland and Malta (Fig. 4e) and, by 2007, to Italy (Fig. 4f). By 2008, several exchanges involved different localities, as shown in Fig. 4g, such as a flow from South Africa to the United Kingdom. In Fig. 4h, by 2009, after the intensification in Italy, the initial phase of the migratory events from Italy to the United Kingdom, from Italy to France, and from the United Kingdom to Slovenia is highlighted. Meanwhile, Fig. 4i mainly depicts the beginning of the dispersal that occurred by 2010 from the United Kingdom to Sweden, the extension of the migratory process from the United Kingdom to Slovenia.

**FIG 5:**
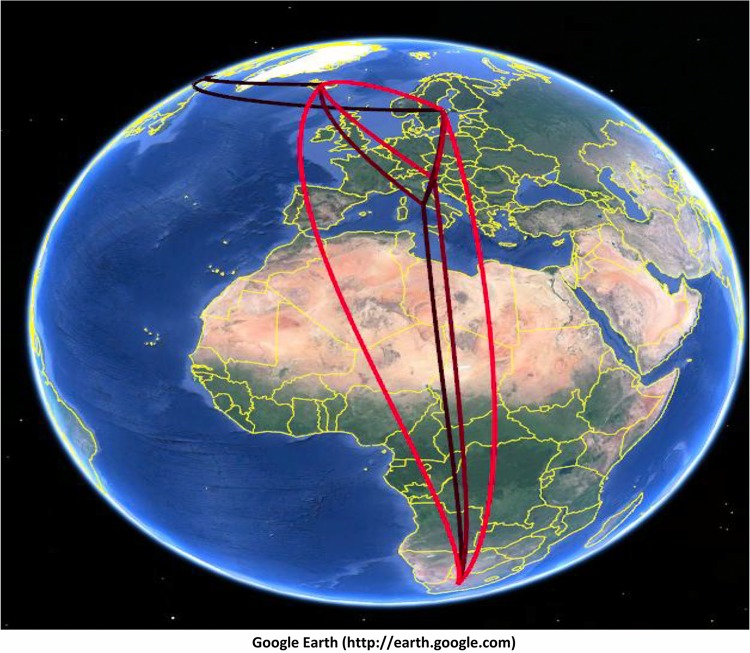
The frequently invoked rates identified by SpreaD (using Bayes factor test) were indicated and reconstructed as a map based on satellite pictures made available in Google Earth (Google Earth Pro V.7.3.2.5491).

By 2011, the strain appeared to have spread to Sweden. By 2012, C:P1.5-1,10-8:F3-6:ST-11(cc11) moved from Italy to France and back to the United Kingdom with a contemporary exchange from the United Kingdom to Slovenia (Fig. 4l). Then, by 2013, an exchange probably occurred from the United Kingdom to Canada. Other projections (Fig. 4m) highlighted the diffusion from France to the United Kingdom by 2014 and from Italy to Slovenia. Figure 4n shows that, by the beginning of 2015, a migration from France to Spain also occurred. In addition, the dispersal patterns that frequently occurred among neighboring European countries involved Italy and Slovenia, Italy and Sweden, and Slovenia and Sweden (Fig. 5). The pathways connecting more remote areas, such as Canada and Europe (Sweden or Iceland), or Iceland with Europe (Italy, Slovenia, and Sweden), were also frequently invoked, in addition to those between Italy and South Africa, Slovenia and South Africa, South Africa and Sweden, and South Africa and Iceland (Fig. 5).

### Phylogeographic analysis of the second subset (Italian and international isolates).

The phylogeographic analysis of the Italian and representative international isolates (“second subset” in Fig. S1 in the supplemental material) suggested an origin of subclade B1 in 2005 (95% HPD, 2002 to 2007) in United Kingdom (sp = 0.95), as found for the whole data set. A number of statistically supported clusters were identified. Focusing only on the main clade (M), the phylogeographic tree suggested a United Kingdom origin (sp = 0.99), with two distinct introductions, corresponding to clades a and b, and suggesting a United Kingdom origin for the Italian isolates. Subclade a was dated to 2008 (95% HPD, 2007 to 2011) and included two isolates from the United Kingdom and six from Italy. The four isolates from the cruise ship outbreak were closely related to each other, and dated to 2011/2012 (95% HPD, 2011 to 2012), as also shown in the phylogeography of the whole data set (Fig. 3).

Subclade b was dated to 2011 (95% HPD, 2009 to 2011) and also likely originated from the United Kingdom (sp = 0.99). This clade included European isolates from the United Kingdom, Ireland, Sweden, and France, which were related to Italian isolates from different regions. All the “Tuscany-outbreak-strain” isolates were included in subclade b (Fig. S1). Many supported internal clusters in subclade b (dating from 2013 to 2015), involving Italian isolates, were identified. Of the Italian isolates, the two ST-2780 Italian meningococci (ID code 142 and 145) appeared to be closely related to two ST-11 Italian meningococci collected in the same time period (2015). Therefore, the “Tuscany outbreak strain” was probably introduced between 2013 and 2014, having originated in the United Kingdom in 2011 (95% HPD, 2009 to 2011) (Fig. S1).

## DISCUSSION

The evidence presented herein offers deepened insight into the epidemiology and phylogeographic spread of the invasive and hypervirulent meningococcus strain C:P1.5-1,10-8:F3-6:ST-11(cc11) and its close relationship to internationally disseminating clones. The analysis of meningococci from Italy and the rest of the world demonstrated both local dispersal and intercountry spread. The “Tuscany-outbreak strain” variant of this particular meningococcus serves as a paradigm for the introduction and spread of a novel hypervirulent organism into a geographic area with an immunologically naïve human population. The combination of epidemiological investigation with phylogenetic analyses allowed the identification of clusters and confirmation of possible transmission links among IMD cases. In particular, cases belonging to the same statistically supported clusters shared the same contact locations and/or risk factors, such as, for example, close contact with men who have sex with men (MSM) (e.g., cases who had two MSM family members with a sexual relationship) (10). Several studies have reported outbreaks of serogroup C meningococci associated with attendance at social venues of high transmission risk such as discos/gay venues (31–35). It is known that crowded conditions promote meningococcal transmission due to behavioral risk factors, including the following: smoking, drug use, alcohol use, and intimate kissing with multiple partners. The phylogenetic analyses allowed the identification of additional clusters that may not have been identified with epidemiological data alone.

The mean evolutionary rate estimated for C:P1.5-1,10-8:F3-6:ST-11(cc11) was 3.7 × 10^−6^ substitutions per site per year (95% HPD, 3.1 × 10^−6^ to 4.4 × 10^−6^) for the whole data set (*n* = 311 isolates) and 3.1 × 10^−6^ substitutions per site per year (95% HPD, 2.4 × 10^−6^ to 3.8 × 10^−6^) for the subset of isolates containing Italian isolates (*n* = 63) and international isolates (*n* = 70). These estimated values were comparable to those reported previously for serogroup A, ST-7, and ST-2859 meningococci, where a mean evolutionary rate of 3.1 × 10^-6^ substitutions per site per year (95% HPD, 2.30 × 10^−6^ to 3.85 × 10^−6^) was estimated for isolates with a putative common ancestor dated to 2000 (95% HPD, 1995 to 2001) (36). Compared to studies of other pathogenic bacteria, the estimated rate was similar to that calculated for Staphylococcus aureus (3 × 10^−6^) (37) and it was higher than the rates estimated for Salmonella enterica serovar Typhimurium (1.9 × 10^−7^ and 3.9 × 10^−7^) (38) and Yersinia pestis (2 × 10^−8^) (39).

The phylogeographic analyses of the C:P1.5-1,10-8:F3-6:ST-11(cc11) N. meningitidis isolates from different countries permitted the construction of a model of their spread. In this model, these meningococci likely originated in the United Kingdom in 1995 (95% HPD, 1992 to 1998), followed by dissemination to other countries. By the end of the 1990s and the beginning of the 2000s, the strain was identified in the United Kingdom, Spain, Iceland, South Africa, Ireland, and Malta. This is consistent with epidemiological data from the United Kingdom, Ireland, Spain, and Iceland, where serogroup C IMD increased in the late 1990s to early 2000s, which can be attributed to these meningococci (40, 41). As a consequence of this increase in serogroup C IMD, the United Kingdom was the first country to introduce immunization with meningococcal C conjugate (MCC) vaccine in 1999 (42). Subsequently, Ireland, Spain in 2000 (43), and Iceland in 2002, introduced mass MCC vaccination campaigns (42).

In Italy, the C:P1.5-1,10-8:F3-6:ST-11(cc11) strain segregated into two distinct clades, both apparently originating from the United Kingdom, but at different time periods. The first introduction in Italy occurred in 2007 (95% HPD, 2002 to 2009) and again in 2011: at that time, this variant was spreading among European countries. The second introduction was between 2013 and 2014, and the outbreak in Tuscany was probably due to this introduction of a United Kingdom strain which originated in 2011 (95% HPD, 2009 to 2011). This strain was distinct from that causing the outbreak affecting a cruise ship approaching the port of Livorno in 2012, which belonged to a distinct cluster. The latter occurred at a different time and probably arose from a different migration route, going extinct before causing further IMD cases in the region.

Before drawing conclusions, limits and possible bias of the study should be mentioned. First of all, this model of epidemic spread is dependent on the genomes available for analysis and is subject to potential sampling bias [for example if the C:P1.5-1,10-8:F3-6:ST-11(cc11) N. meningitidis isolates are poorly represented in the isolate collections examined]. This could lead to the failure to identify some intermediate steps in transmission although, given the hypervirulent nature of this particular meningococcus, this is perhaps unlikely. Second, this model is based on IMD surveillance and does not take into account the frequency distribution of carriage, which is however assumed to be relatively short for such hypervirulent strains.

The genetic diversity of meningococci, which is related to the diversity observed in the epidemiology of IMD (6), gives epidemiological surveillance combined with high-resolution molecular characterization a central role in IMD control and prevention. This is especially important in tracking the spread of the hypervirulent meningococcal cc11, members of which have a long history of causing disease outbreaks, often involving multiple countries or continents (44). The phylogeographic analysis of C:cc11 isolates from Italy, in the context of those from other countries, demonstrated that after multiple introductions from abroad, closely related genotypes segregated into distinct clades and clusters, consistent with the circulation of different C:cc11 variants, which caused IMD cases at different times. In particular, the Tuscany outbreak represented a paradigmatic event of clonal amplification of one specific variant, C:P1.5-1,10-8:F3-6:ST-11(cc11), resulting from the sustained transmission of this hypervirulent meningococcus after it was introduced into a (presumably) immunologically naïve geographically restricted population. This strain, which was likely introduced in 2014, differed from other related yet distinct meningococci that caused smaller outbreaks in the same region.

In conclusion, these data show how WGS analysis combined with phylogeography, enables the identification of clusters, characterization of phylogenetic relationships, and tracking the dissemination of strains, all of which are invaluable in understanding IMD epidemic dynamics and in the planning of successful public health interventions.

## Supplementary Material

Supplemental file 1

## References

[1] Trotter CL, Gay NJ, Edmunds WJ 2006 The natural history of meningococcal carriage and disease. Epidemiol Infect 134:556–566. doi:10.1017/S0950268805005339. 16238823 PMC2870423

[2] Harrison OB, Claus H, Jiang Y, Bennett JS, Bratcher HB, Jolley KA, Corton C, Care R, Poolman JT, Zollinger WD, Frasch CE, Stephens DS, Feavers I, Frosch M, Parkhill J, Vogel U, Quail MA, Bentley SD, Maiden MC 2013 Description and nomenclature of Neisseria meningitidis capsule locus. Emerg Infect Dis 19:566–573. doi:10.3201/eid1904.111799. 23628376 PMC3647402

[3] Stephens DS 1999 Uncloaking the meningococcus: dynamics of carriage and disease. Lancet 353:941–942. doi:10.1016/S0140-6736(98)00279-7. 10459897

[4] Maiden MCJ, Bygraves JA, Feil E, Morelli G, Russell JE, Urwin R, Zhang Q, Zhou J, Zurth K, Caugant DA, Feavers IM, Achtman M, Spratt BG 1998 Multilocus sequence typing: a portable approach to the identification of clones within populations of pathogenic microorganisms. Proc Natl Acad Sci U S A 95:3140–3145. doi:10.1073/pnas.95.6.3140. 9501229 PMC19708

[5] Yazdankhah SP, Kriz P, Tzanakaki G, Kremastinou J, Kalmusova J, Musilek M, Alvestad T, Jolley KA, Wilson DJ, McCarthy ND, Caugant DA, Maiden MC 2004 Distribution of serogroups and genotypes among disease-associated and carried isolates of *Neisseria meningitidis* from the Czech Republic, Greece, and Norway. J Clin Microbiol 42:5146–5153. doi:10.1128/JCM.42.11.5146-5153.2004. 15528708 PMC525265

[6] Caugant DA, Maiden MC 2009 Meningococcal carriage and disease - population biology and evolution. Vaccine 27(Suppl 2):B64–B70. doi:10.1016/j.vaccine.2009.04.061. 19464092 PMC2719693

[7] ISS. 2018 Report Sorveglianza delle malattie batteriche invasive in Italia. http://old.iss.it/binary/mabi/cont/Report2017.pdf. Accessed 10 December 2018.

[8] Italian Ministry of Health. 2014 National Vaccination Plan 2012 − 2014. Italian Ministry of Health, Rome, Italy. (In Italian.) http://www.salute.gov.it/imgs/C_17_pubblicazioni_1721_allegato.pdf.

[9] Stefanelli P, Miglietta A, Pezzotti P, Fazio C, Neri A, Vacca P, Voller F, D’Ancona FP, Guerra R, Iannazzo S, Pompa MG, Rezza G 2016 Increased incidence of invasive meningococcal disease of serogroup C / clonal complex 11, Tuscany, Italy, 2015 to 2016. Euro Surveill 21(12):pii=30176 10.2807/1560-7917.ES.2016.21.12.30176. 27035155

[10] Miglietta A, Fazio C, Neri A, Pezzotti P, Innocenti F, Azzari C, Rossolini GM, Moriondo M, Nieddu F, Iannazzo S, D’Ancona F, Maraglino FP, Guerra R, Rezza G, Voller F, Stefanelli P 2018 Interconnected clusters of invasive meningococcal disease due to *Neisseria meningitidis* serogroup C ST-11 (cc11), involving bisexuals and men who have sex with men, with discos and gay-venues hotspots of transmission, Tuscany, Italy, 2015 to 2016. Euro Surveill 23(34):pii=1700636 10.2807/1560-7917.ES.2018.23.34.1700636. PMC6113744 30153883

[11] Fazio C, Neri A, Tonino S, Carannante A, Caporali MG, Salmaso S, Mastrantonio P, Stefanelli P 2009 Characterisation of Neisseria meningitidis C strains causing two clusters in the north of Italy in 2007 and 2008. Euro Surveill 14(16):pii=19179 10.2807/ese.14.16.19179-en. 19389338

[12] Stefanelli P, Fazio C, Neri A, Ciammaruconi A, Balocchini E, Anselmo A, Azzari C, Rossolini GM, Vacca P, Fortunato A, Palozzi A, Fillo S, Lista F, Moriondo M, Nieddu F, Rezza G 2016 Genome-based study of a spatio-temporal cluster of invasive meningococcal disease due to *Neisseria meningitidis* serogroup C, clonal complex 11. J Infect 73:136–144. doi:10.1016/j.jinf.2016.05.003. 27235364

[13] Jolley KA, Brehony C, Maiden MC 2007 Molecular typing of meningococci: recommendations for target choice and nomenclature. FEMS Microbiol Rev 31:89–96. doi:10.1111/j.1574-6976.2006.00057.x. 17168996

[14] Jolley KA, Maiden MC 2010 BIGSdb: scalable analysis of bacterial genome variation at the population level. BMC Bioinformatics 11:595. doi:10.1186/1471-2105-11-595. 21143983 PMC3004885

[15] Pekka M, Baldwin A, Hanage WP, Dowson C, Mahenthiralingam E, Corander J 2008 Bayesian modeling of recombination events in bacterial populations. BMC Bioinformatics 9:421. doi:10.1186/1471-2105-9-421. 18840286 PMC2579306

[16] Croucher NJ, Harris SR, Fraser C, Quail MA, Burton J, van der Linden M, McGee L, von Gottberg A, Song JH, Ko KS, Pichon B, Baker S, Parry CM, Lambertsen LM, Shahinas D, Pillai DR, Mitchell TJ, Dougan G, Tomasz A, Klugman KP, Parkhill J, Hanage WP, Bentley SD 2011 Rapid pneumococcal evolution in response to clinical interventions. Science 331:430–434. doi:10.1126/science.1198545. 21273480 PMC3648787

[17] Méric G, Miragaia M, de Been M, Yahara K, Pascoe B, Mageiros L, Mikhail J, Harris LG, Wilkinson TS, Rolo J, Lamble S, Bray JE, Jolley KA, Hanage WP, Bowden R, Maiden MC, Mack D, de Lencastre H, Feil EJ, Corander J, Sheppard SK 2015 Ecological overlap and horizontal gene transfer in *Staphylococcus aureus* and *Staphylococcus epidermidis*. Genome Biol Evol 16:1313–1328. doi:10.1093/gbe/evv066. PMC4453061 25888688

[18] Marttinen P, Hanage WP, Croucher NJ, Connor TR, Harris SR, Bentley SD, Corander J 2012 Detection of recombination events in bacterial genomes from large population segments. Nucleic Acids Res 40:e6. doi:10.1093/nar/gkr928. 22064866 PMC3245952

[19] Tamura K, Stecher G, Peterson D, Filipski A, Kumar S 2013 MEGA6: Molecular Evolutionary Genetics Analysis version 6.0. Mol Biol Evol 30:2725–2729. doi:10.1093/molbev/mst197. 24132122 PMC3840312

[20] Posada D 2008 jModelTest: phylogenetic model averaging. Mol Biol Evol 25:1253–1256. doi:10.1093/molbev/msn083. 18397919

[21] Darriba D, Taboada GL, Doallo R, Posada D 2012 jModelTest 2: more models, new heuristics and parallel computing. Nat Methods 9:772. doi:10.1038/nmeth.2109. PMC4594756 22847109

[22] Strimmer K, von Haeseler A 1997 Likelihood-mapping: a simple method to visualize phylogenetic content of a sequence alignment. Proc Natl Acad Sci U S A 94:6815–6819. doi:10.1073/pnas.94.13.6815. 9192648 PMC21241

[23] Xia X, Xie Z, Salemi M, Chen L, Wang Y 2003 An index of substitution saturation and its application. Mol Phylogenet Evol 26:1–7. doi:10.1016/S1055-7903(02)00326-3. 12470932

[24] Nguyen LT, Schmidt HA, von Haeseler A, Minh BQ 2015 IQ-TREE: a fast and effective stochastic algorithm for estimating maximum likelihood phylogenies. Mol Biol Evol 32:268–274. doi:10.1093/molbev/msu300. 25371430 PMC4271533

[25] Huelsenbeck JP, Ronquist F 2001 MRBAYES: Bayesian inference of phylogenetic trees. Bioinformatics 17:754–755. doi:10.1093/bioinformatics/17.8.754. 11524383

[26] Rambaut A, Lam TT, Carvalho LM, Pybus OG 2016 Exploring the temporal structure of heterochronous sequences using TempEst. Virus Evol 2:vew007. doi:10.1093/ve/vew007. 27774300 PMC4989882

[27] Drummond AJ, Rambaut A 2007 BEAST: Bayesian evolutionary analysis by sampling trees. BMC Evol Biol 7:214. doi:10.1186/1471-2148-7-214. 17996036 PMC2247476

[28] Kass RE, Raftery AE 1995 Bayes factors. J Am Stat Assoc 90:773–795. doi:10.2307/2291091.

[29] Bielejec F, Rambaut A, Suchard MA, Lemey P 2011 SPREAD: Spatial Phylogenetic Reconstruction of Evolutionary Dynamics. Bioinformatics 27:2910–2912. doi:10.1093/bioinformatics/btr481. 21911333 PMC3187652

[30] Stefanelli P, Fazio C, Neri A, Isola P, Sani S, Marelli P, Martinelli C, Mastrantonio P, Pompa MG 2012 Cluster of invasive Neisseria meningitidis infections on a cruise ship, Italy, October 2012. Euro Surveill 17(50):pii=20336 10.2807/ese.17.50.20336-en. 23241233

[31] Hauri AM, Ehrhard I, Frank U, Ammer J, Fell G, Hamouda O, Petersen L 2000 Serogroup C meningococcal disease outbreak associated with discotheque attendance during carnival. Epidemiol Infect 124:69–73. doi:10.1017/s0950268899003416. 10722132 PMC2810885

[32] Riesbeck K, Orvelid-Mölling P, Fredlund H, Olcén P 2000 Long-term persistence of a discotheque-associated invasive Neisseria meningitidis group C strain as proven by pulsed-field gel electrophoresis and porA gene sequencing. J Clin Microbiol 38:1638–1640. 10747157 10.1128/jcm.38.4.1638-1640.2000 PMC86509

[33] Marcus U, Vogel U, Schubert A, Claus H, Baetzing-Feigenbaum J, Hellenbrand W, Wichmann O 2013 A cluster of invasive meningococcal disease in young men who have sex with men in Berlin, October 2012 to May 2013. Euro Surveill 18(28):pii=20523 10.2807/1560-7917.ES2013.18.28.20523. 23870095

[34] European Centre for Disease Prevention and Control (ECDC). 2013 Invasive meningococcal disease among men who have sex with men. Rapid risk assessment. European Centre for Disease Prevention and Control, Stockholm, Sweden.

[35] Aubert L, Taha M, Boo N, Le Strat Y, Deghmane AE, Sanna A, Barret AS, Lévy-Bruhl D, Vandentorren S, Parent Du Châtelet I 2015 Serogroup C invasive meningococcal disease among men who have sex with men and in gay-oriented social venues in the Paris region: July 2013 to December 2014. Euro Surveill 20(3):pii=21016 10.2807/1560-7917.ES2015.20.3.21016. 25635319

[36] Lamelas A, Harris SR, Röltgen K, Dangy JP, Hauser J, Kingsley RA, Connor TR, Sie A, Hodgson A, Dougan G, Parkhill J, Bentley SD, Pluschke G 2014 Emergence of a new epidemic *Neisseria meningitidis* serogroup A clone in the African meningitis belt: high-resolution picture of genomic changes that mediate immune evasion. mBio 21:e01974-14. doi:10.1128/mBio.01974-14. PMC4212839 25336458

[37] Harris SR, Feil EJ, Holden MT, Quail MA, Nickerson EK, Chantratita N, Gardete S, Tavares A, Day N, Lindsay JA, Edgeworth JD, de Lencastre H, Parkhill J, Peacock SJ, Bentley SD 2010 Evolution of MRSA during hospital transmission and intercontinental spread. Science 327:469–474. doi:10.1126/science.1182395. 20093474 PMC2821690

[38] Okoro CK, Kingsley RA, Connor TR, Harris SR, Parry CM, Al-Mashhadani MN, Kariuki S, Msefula CL, Gordon MA, de Pinna E, Wain J, Heyderman RS, Obaro S, Alonso PL, Mandomando I, MacLennan CA, Tapia MD, Levine MM, Tennant SM, Parkhill J, Dougan G 2012 Intracontinental spread of human invasive *Salmonella typhimurium* pathovariants in sub-Saharan Africa. Nat Genet 44:1215–1221. doi:10.1038/ng.2423. 23023330 PMC3491877

[39] Morelli G, Song Y, Mazzoni CJ, Eppinger M, Roumagnac P, Wagner DM, Feldkamp M, Kusecek B, Vogler AJ, Li Y, Cui Y, Thomson NR, Jombart T, Leblois R, Lichtner P, Rahalison L, Petersen JM, Balloux F, Keim P, Wirth T, Ravel J, Yang R, Carniel E, Achtman M 2010 *Yersinia pestis* genome sequencing identifies patterns of global phylogenetic diversity. Nat Genet 42:1140–1143. doi:10.1038/ng.705. 21037571 PMC2999892

[40] Harrison LH, Trotter CL, Ramsay ME 2009 Global epidemiology of meningococcal disease. Vaccine 24:B51–B63. doi:10.1016/j.vaccine.2009.04.063. 19477562

[41] Trotter CL, Chandra M, Cano R, Larrauri A, Ramsay ME, Brehony C, Jolley KA, Maiden MC, Heuberger S, Frosch M 2007 A surveillance network for meningococcal disease in Europe. FEMS Microbiol Rev 31:27–36. doi:10.1111/j.1574-6976.2006.00060.x. 17168995

[42] Campbell H, Borrow R, Salisbury D, Miller E 2009 Meningococcal C conjugate vaccine: the experience in England and Wales. Vaccine 27:B20–B29. doi:10.1016/j.vaccine.2009.04.067. 19477053

[43] Cano R, Larrauri A, Mateo S, Alcalá B, Salcedo C, Vázquez JA 2004 Impact of the meningococcal C conjugate vaccine in Spain: an epidemiological and microbiological decision. Euro Surveill 9(7):11–15. doi:10.2807/esm.09.07.00474-en. 15318008

[44] Lucidarme J, Hill DM, Bratcher HB, Gray SJ, Du Plessis M, Tsang RS, Vazquez JA, Taha MK, Ceyhan M, Efron AM, Gorla MC, Findlow J, Jolley KA, Maiden MC, Borrow R 2015 Genomic resolution of an aggressive, widespread, diverse and expanding meningococcal serogroup B, C and W lineage. J Infect 71:544–552. doi:10.1016/j.jinf.2015.07.007. 26226598 PMC4635312

