# Supplementary material for: Reconstruction of Dispersal Patterns of Hypervirulent Meningococcal Strains of Serogroup C:cc11 by Phylogenomic Time Trees
Source: J Clin Microbiol. 2019 Dec 23;58(1):e01351-19. doi: 10.1128/JCM.01351-19 (PMC6935922; doi:10.1128/JCM.01351-19)
Supplement: Supplemental file 1 [file JCM.01351-19-s0001.pdf]

Table S1. Detailed information of *Neisseria meningitidis* whole dataset.

Tip label	ID (http://pubmlst.org/Neisseria)	Country	Date onset	Year
1	20155	UK		2010
2	20329	UK		2011
3	20359	UK		2011
4	21185	Malta		2011
5	21232	UK		2011
6	21253	UK		2011
7	21359	UK		2012
8	21364	UK		2012
9	21407	UK		2012
10	26733	France		2013
11	26821	France		2013
12	28083	UK		2012
13	28085	UK		2012
14	28093	UK		2013
15	28094	UK		2013
16	28097	UK		2013
17	28099	UK		2013
18	28100	UK		2013
19	28103	UK		2013
20	28104	UK		2013
21	28105	UK		2013
22	29572	UK		2004
23	29574	UK		2004
24	29580	UK		2004
25	29581	UK		2004
26	29582	UK		2004
27	29583	UK		2004
28	29584	UK		2004
29	29585	UK		2004
30	29586	UK		2004
31	29587	UK		2004
32	29588	Ireland		2004
33	29594	UK		2004
34	29595	UK		2004
35	29599	UK		2005
36	29601	UK		2005
37	29602	UK		2005
38	29603	Malta		2005
39	29604	UK		2005

40	29607	Ireland		2005
41	29614	UK		2006
42	29618	Ireland		2006
43	29619	UK		2006
44	29621	UK		2006
45	29624	UK		2006
46	29627	UK		2007
47	29631	UK		2007
48	29636	UK		2007
49	29637	UK		2007
50	29644	UK		1998
51	29654	UK		1999
52	29658	UK		1999
53	29660	UK		1999
54	29663	UK		2000
55	29664	UK		2000
56	29667	UK		2000
57	29668	UK		2000
58	29685	UK		2002
59	29701	UK		2003
60	29750	UK		1998
61	29751	UK		1998
62	29752	UK		1998
63	29841	UK		2000
64	29851	UK		2001
65	29855	UK		2001
66	29856	UK		2001
67	29857	UK		2001
68	29862	UK		2002
69	29866	UK		2002
70	29867	UK		2002
71	29869	UK		2002
72	29872	UK		2002
73	29873	UK		2002
74	29874	UK		2003
75	29882	UK		2008
76	29884	UK		2009
77	29885	UK		2009
78	29886	UK		2008
79	29887	UK		2008
80	29890	UK		2010
81	29892	UK		2010
82	29894	UK		2009
83	29897	UK		2013

84	29903	UK		1998
85	29906	UK		1998
86	29910	UK		1998
87	29913	UK		1999
88	29915	UK		1999
89	29917	UK		1999
90	29919	UK		1999
91	29921	UK		1999
92	29925	UK		1999
93	29972	UK		2003
94	29974	UK		2003
95	29977	UK		2003
96	30126	Spain		2001
97	30127	Spain		2001
98	30130	Spain		1999
99	30131	Spain		2001
100	30132	Spain		2001
101	30133	Spain		2001
102	30134	Spain		2002
103	30194	UK		1999
104	30195	UK		1999
105	30197	UK		1999
106	30198	UK		1999
107	30199	UK		1999
108	30200	UK		1999
109	30201	UK		1999
110	30204	UK		1999
111	30205	UK		1999
112	30206	UK		1999
113	30207	UK		1999
114	30208	UK		1999
115	30209	UK		2013
116	30210	UK		2013
117	30698	Ireland		2014
118	34545	Canada		
119	34744	France		2014
120	35650	UK		2014
121	35693	UK		2014
122▲	36386	Italy	22/01/2015	2015
123#	36444	Italy	07/10/2012	2012
124#	36446	Italy	07/10/2012	2012
125#	36447	Italy	07/10/2012	2012
126▲	36448	Italy	07/02/2015	2015
127	36449	Italy	22/02/2015	2015

128▲	36450	Italy	20/03/2015	2015
129▲	36451	Italy	06/01/2015	2015
130▲	36452	Italy	20/01/2015	2015
131▲	36453	Italy	15/02/2015	2015
132	36460	Italy	01/11/2012	2012
133	36461	Italy	03/04/2014	2014
134	36466	Italy	21/08/2012	2012
135	36467	Italy	01/03/2012	2012
136	36468	Italy	11/02/2012	2012
137	36469	Italy	03/04/2012	2012
138	36470	Italy	02/03/2012	2012
139	36471	Italy	21/04/2012	2012
140	36472	Italy	28/04/2012	2012
141	36473	Italy	23/10/2012	2012
142▲	36670	Italy	25/04/2015	2015
143▲	36765	Italy	16/06/2014	2014
144	36766	Italy	12/04/2014	2014
145▲	36767	Italy	11/05/2015	2015
146▲	36768	Italy	06/05/2015	2015
147▲	36769	Italy	06/02/2015	2015
148▲	36770	Italy	15/02/2015	2015
149	36771	Italy	20/01/2015	2015
150	36772	Italy	26/11/2014	2014
151	36773	Italy	09/11/2014	2014
152	36774	Italy	12/07/2014	2014
153	36775	Italy	09/06/2014	2014
154	36776	Italy	06/06/2014	2014
155	36777	Italy	24/04/2014	2014
156	36782	Italy	22/01/2015	2015
157▲	36784	Italy	08/06/2015	2015
158	36821	Ireland		2014
159	36823	Ireland		2015
160	37019	Italy	14/03/2014	2014
161	37020	Italy	17/08/2014	2015
162	37593	Italy	05/02/2013	2013
163	37594	Italy	25/04/2013	2013
164	37595	Italy	07/08/2013	2013
165	37596	Italy	29/09/2013	2013
166	37597	Italy	13/01/2014	2014
167	37598	Italy	18/01/2014	2014
168	37599	Italy	28/12/2013	2013
169	37600	Italy	14/11/2013	2013
170	37601	Italy	24/02/2014	2014
171	37602	Italy	09/12/2013	2013

172	37679	UK		2014
173	37752	UK		2014
174	37758	UK		2014
175	37763	UK		2014
176	37816	UK		2015
177	37894	UK		2015
178	37918	UK		2015
179	38153	Slovenia		2012
180	38155	Slovenia		2013
181	38159	Slovenia		2014
182	38161	Slovenia		2015
183▲	38838	Italy	29/08/2015	2015
184▲	38839	Italy	30/07/2015	2015
185	38847	Italy	14/01/2013	2013
186	38848	Italy	06/03/2013	2013
187	38849	Italy	05/03/2013	2013
188	38867	Italy	13/12/2013	2013
189	38871	Italy	02/03/2014	2014
190	39319	UK		2015
191	39353	UK		2015
192	39361	UK		2015
193	39388	UK		2015
194	39403	UK		2015
195	39409	UK		2015
196▲	39569	Italy	02/10/2015	2015
197	39940	France		2015
198	39941	France		2015
199	40237	France		2015
200	40278	France		2015
201	40388	France		2015
202	40389	Spain		2015
203	40395	France		2015
204	40396	France		2015
205	40410	France		2016
206	40485	France		2015
207	40540	South Africa		2002
208	40545	South Africa		2002
209	40547	South Africa		2005
210	40574	South Africa		2006
211	40575	South Africa		2006
212	40605	South Africa		2003
213	40608	South Africa		2007
214	40610	South Africa		2007
215	40615	South Africa		2003

216	40624	South Africa		2007
217	40641	South Africa		2004
218	40643	South Africa		2004
219	40647	South Africa		2008
220	40654	South Africa		2008
221	40665	South Africa		2008
222	40703	South Africa		2010
223	40706	South Africa		2010
224	40709	South Africa		2010
225	40716	South Africa		2011
226	40738	South Africa		2012
227	41232	France		2015
228	41593	France		2015
229	41614	Italy	26/04/2015	2015
230▲	41642	Italy	24/11/2015	2015
231	41646	Italy	21/08/2015	2015
232▲	41648	Italy	01/10/2015	2015
233▲	41659	Italy	07/01/2016	2016
234▲	41661	Italy	12/01/2016	2016
235▲	41665	Italy	05/01/2016	2016
236▲	41667	Italy	30/01/2016	2016
237▲	41668	Italy	03/02/2016	2016
238	41673	France		2016
239▲	41678	Italy	01/10/2015	2015
240	41695	Slovenia		2015
241	41732	France		2016
242	41794	France		2016
243▲	42032	Italy	05/02/2016	2016
244	42034	Italy	24/02/2016	2016
245▲	42035	Italy	04/03/2016	2016
246	42037	France		2016
247	42190	France		2016
248	42192	France		2016
249	42375	Sweden		2014
250	42382	Sweden		2014
251	42383	Sweden		2014
252	42390	Sweden		2014
253	42540	UK		2016
254	42541	UK		2016
255	42597	UK		2016
256	42766	France		2016
257	42768	France		2016
258	42772	France		2016
259	42781	France		2016

260	42788	Italy	25/03/2016	2016
261▲	42863	Italy	25/02/2016	2016
262	42864	Italy	07/03/2016	2016
263	42865	Italy	23/03/2016	2016
264	43926	France		2016
265	43989	France		2016
266	43997	France		2016
267	43998	France		2016
268	44779	UK		2016
269	44824	UK		2016
270	44846	Iceland		2002
271	44863	Iceland		2004
272	44864	Iceland		2004
273	44874	Iceland		2005
274▲	44976	Italy	17/06/2016	2016
275	44982	Sweden		2016
276	45334	France		2016
277	45349	France		2016
278	47308	UK		2016
279	49261	Italy	05/08/2016	2016
280▲	49275	Italy	18/04/2016	2016
281	49276	Italy	19/05/2016	2016
282▲	49277	Italy	26/05/2016	2016
283	49278	Italy	12/06/2016	2016
284	49280	Italy	26/07/2016	2016
285▲	49281	Italy	28/07/2016	2016
286▲	50946	Italy	14/07/2016	2016
287	50947	Italy	27/10/2016	2016
288▲	50948	Italy	26/02/2016	2016
289▲	50949	Italy	01/11/2016	2016
290	52812	Italy	18/03/2014	2014
291	52933	Italy	28/04/2016	2016
292	52934	Italy	06/05/2016	2016
293▲	52947	Italy	26/11/2016	2016
294	52952	Italy	29/08/2016	2016
295	53090	UK		2016
296	53100	UK		2016
297	53276	UK		2017
298	53304	UK		2017
299	53573	Italy	10/11/2016	2016
300	53574	Italy	10/01/2017	2017
301▲	53575	Italy	03/02/2017	2017
302	53576	Italy	31/12/2016	2016
303	53577	Italy	08/02/2017	2017

304	53578	Italy	17/02/2017	2017
305	53713	Italy	18/06/2014	2014
306▲	53714	Italy	21/04/2015	2015
307▲	53715	Italy	29/06/2016	2016
308▲	53717	Italy	06/03/2017	2017
309	53718	Italy	18/04/2017	2017
310	53719	Italy	17/04/2017	2017
311#	36445	Italy	07/10/2012	2012

#: isolates from port of Livorno (Tuscany)

▲: isolates belonging to "*Tuscany-outbreak strain*"

Table S2. Formal model selection (Bayes Factor tests) to determine the most appropriate model for the analysis

Bayes Factors tests indicated that the data best fit the relaxed molecular clock (2lnBF between the strict and relaxed clock was 116.712 in favor of the second) and the BSP was better than other demographic models (2lnBF > 117).

Table S3. Xia's test for substitution saturation: testing whether the observed index of substitution saturation (Iss) is significantly lower than the critical value of index of substitution saturation (Iss.c) in the *N. meningitidis* core genome SNPs alignment, whole dataset. Analysis performed on fully resolved sites only. Iss Sym is Iss.c assuming a symmetrical topology. Iss Asym is Iss.c assuming an asymmetrical topology. This test ($p < 0.001$) indicated that the Iss was significantly lower than the Iss c, confirming the dataset has not experienced substitution saturation and was suitable for further phylogenetic analysis

NumOTU	Iss	Iss.cSym	T	DF	P	Iss.cAsym	T	DF	P
4	0.027	0.787	94.899	386	0.0000	0.756	91.040	386	0.0000
8	0.028	0.741	87.747	386	0.0000	0.630	74.084	386	0.0000
16	0.029	0.699	89.007	386	0.0000	0.490	61.263	386	0.0000
32	0.031	0.690	93.490	386	0.0000	0.361	46.784	386	0.0000

Note: two-tailed tests are used.

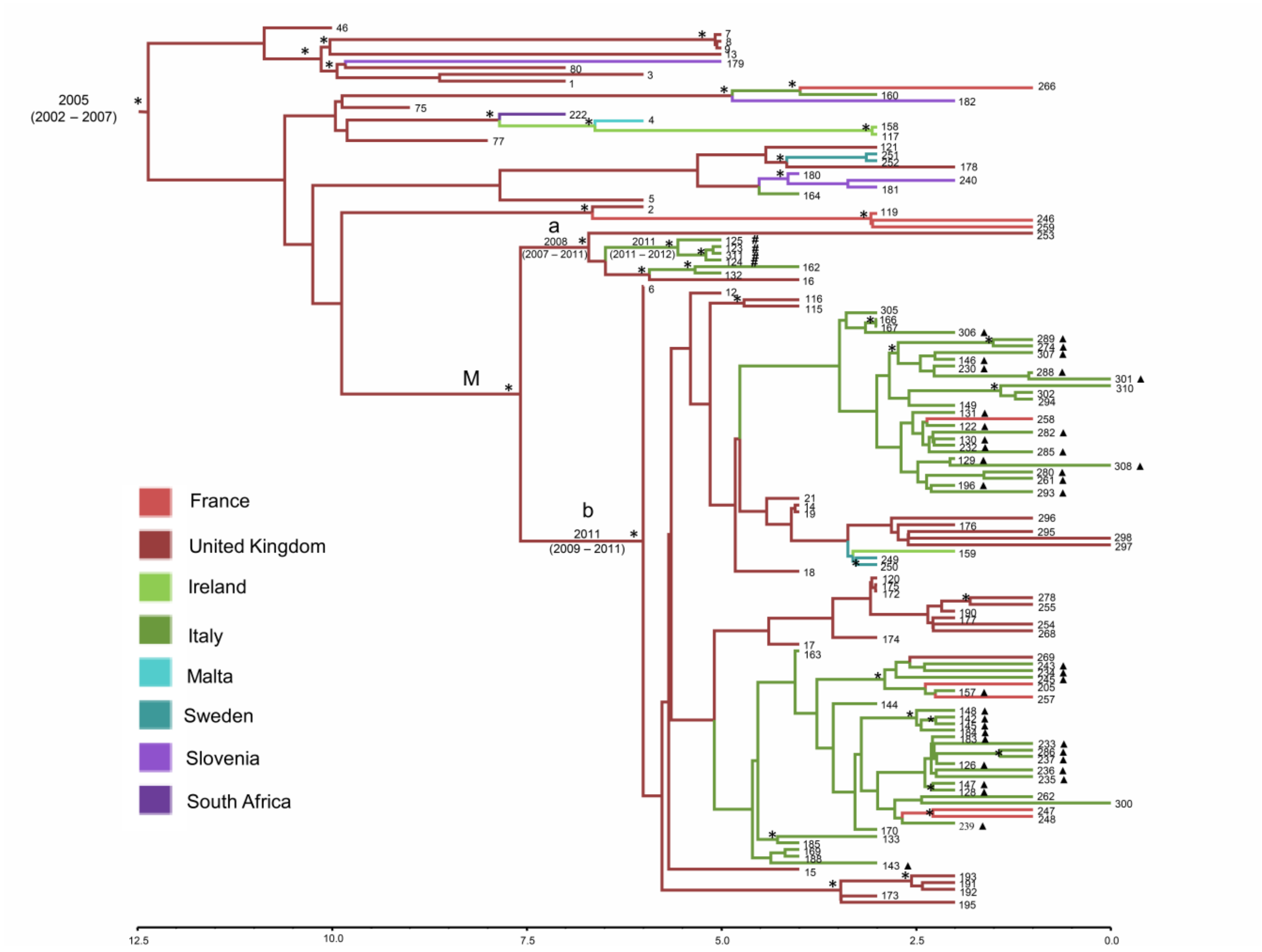

Fig. S1. **Bayesian phylogeographic tree of *N. meningitidis* (cc11) strains of the second subset.** The scale axis below the tree showed the time (years) before the present. Main statistically supported clades and clusters were indicated. The asterisk (*) along the branch represents significant statistical support for the clade subtending that branch (posterior probability > 90%). *The* # symbol next to the tips indicates the four isolates from cases occurred in a cruise ship docked to the port of Livorno. A full triangle symbol next to the tips indicates “*Tuscany-outbreak-strains”*. Geographic locations were shown with different colors in the tree and reported as legend.
